# Small molecular inhibitors for KRAS-mutant cancers

**DOI:** 10.3389/fimmu.2023.1223433

**Published:** 2023-08-18

**Authors:** Xuan Wu, Wenping Song, Cheng Cheng, Ziyang Liu, Xiang Li, Yu Cui, Yao Gao, Ding Li

**Affiliations:** ^1^ Department of Internal Medicine, The Affiliated Cancer Hospital of Zhengzhou University and Henan Cancer Hospital, Zhengzhou, China; ^2^ Academy of Medical Science, Zhengzhou University, Zhengzhou, China; ^3^ Department of Pharmacy, The Affiliated Cancer Hospital of Zhengzhou University and Henan Cancer Hospital, Zhengzhou, China; ^4^ Henan Engineering Research Center for Tumor Precision Medicine and Comprehensive Evaluation, Henan Cancer Hospital, Zhengzhou, China; ^5^ Henan Provincial Key Laboratory of Anticancer Drug Research, Henan Cancer Hospital, Zhengzhou, China

**Keywords:** RAS mutation, different isoform, RAS inhibitor, immunotherapy, combination strategy

## Abstract

Three rat sarcoma (RAS) gene isoforms, KRAS, NRAS, and HRAS, constitute the most mutated family of small GTPases in cancer. While the development of targeted immunotherapies has led to a substantial improvement in the overall survival of patients with non-KRAS-mutant cancer, patients with RAS-mutant cancers have an overall poorer prognosis owing to the high aggressiveness of RAS-mutant tumors. KRAS mutations are strongly implicated in lung, pancreatic, and colorectal cancers. However, RAS mutations exhibit diverse patterns of isoforms, substitutions, and positions in different types of cancers. Despite being considered “undruggable”, recent advances in the use of allele-specific covalent inhibitors against the most common mutant form of RAS in non-small-cell lung cancer have led to the development of effective pharmacological interventions against RAS-mutant cancer. Sotorasib (AMG510) has been approved by the FDA as a second-line treatment for patients with KRAS-G12C mutant NSCLC who have received at least one prior systemic therapy. Other KRAS inhibitors are on the way to block KRAS-mutant cancers. In this review, we summarize the progress and promise of small-molecule inhibitors in clinical trials, including direct inhibitors of KRAS, pan-RAS inhibitors, inhibitors of RAS effector signaling, and immune checkpoint inhibitors or combinations with RAS inhibitors, to improve the prognosis of tumors with RAS mutations.

## 1 Introduction

Rat sarcoma (RAS) genes have been recognized as the major oncogenes undergoing mutation for several decades (1, 2). Among the three isoforms (KRAS, NRAS, and HRAS), Kirsten rat sarcoma viral oncogene homolog (KRAS) is the common oncogene in a large percentage of cancers, including pancreatic cancer, non-small cell lung cancer (NSCLC), and colorectal cancer (3–6). Mutations in RAS lead to the dysfunction of its small GTPase activity, preventing it from properly breaking down GTP. The molecule remains in a constant active state that triggers downstream pathways, including the mitogen-activated protein kinase (MAPK) and phosphatidylinositol 3-kinase (PI3K) pathways, leading to oncogenesis.

Attempts to develop effective agents that inhibit RAS mutations have been a failure for a long time (7, 8). In recent years, with the discovery of a new binding site beneath the effector binding switch-II region in RAS protein, several small-molecule agents targeting the KRAS-G12C single-nucleotide mutation (glycine-to-cysteine substitution at codon 12) have been developed and have shown promising efficacy in clinical trials (9–12). Sotorasib (AMG510) has been approved by the FDA as a second-line treatment for patients with KRAS-G12C mutant NSCLC who have received at least one prior systemic therapy (13, 14). Given that several excellent reviews have summarized the role of RAS signaling in oncogenesis and the advances in RAS inhibitors for anti-tumor therapy, we herein focus on KRAS mutations and summarize the promising new treatment options.

## 2 RAS mutations in human cancers

**Figure 1:**
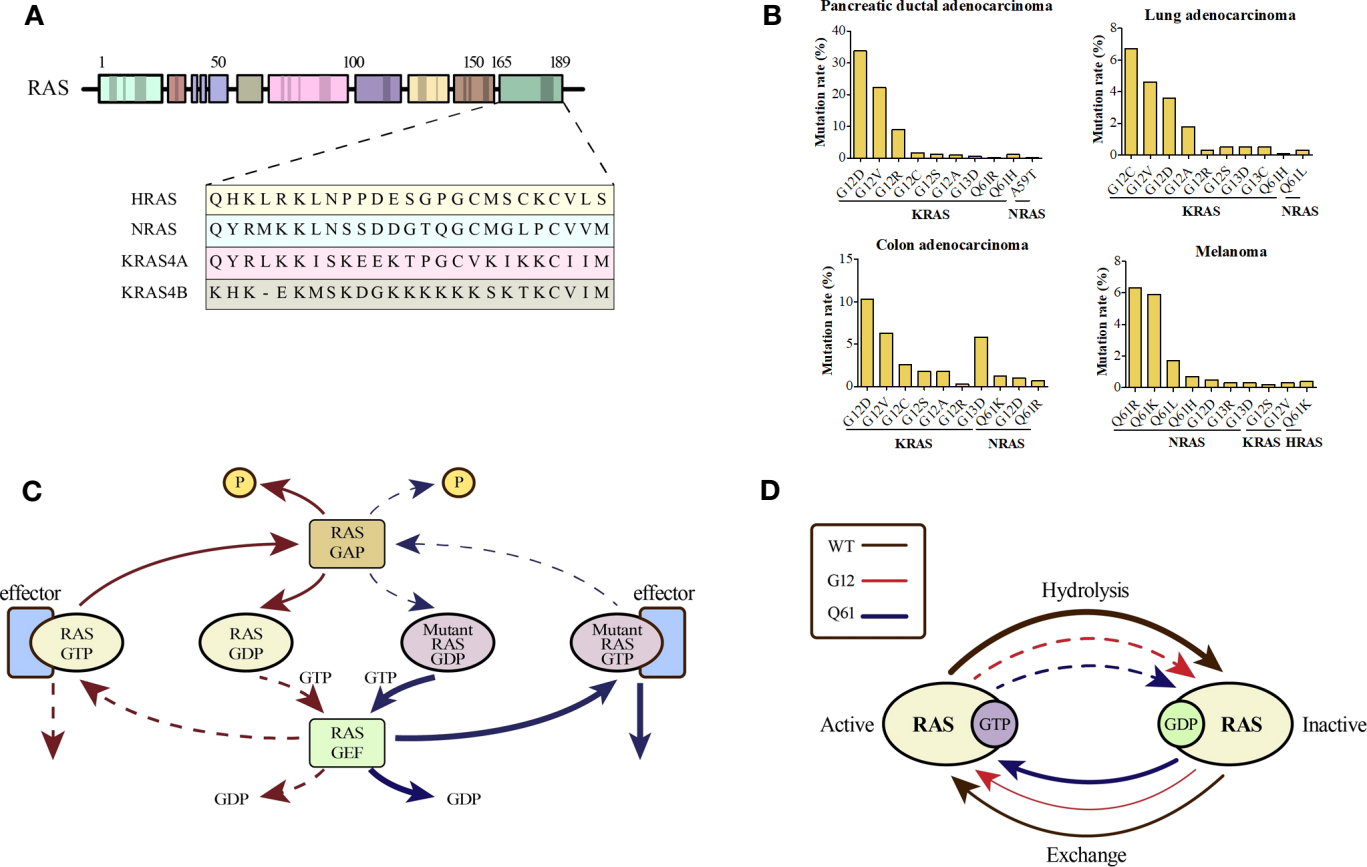
**(A)** An alignment of the carboxy terminus of the three RAS isoforms is shown. The RAS subtypes are highly conserved (~90%) with respect to the entire amino terminal GTPase domain (amino acids 1–166), which contains the GTP-GDP binding site and the interaction site of the effector protein; however, the carboxy terminal part differs and is called the hypervariable zone. **(B)** Percentages of KRAS mutations in codon 12 and NRAS mutations in codon 61 by tissue type for common cancers. **(C)** The canonical nature of RAS is characteristic of a small GTPase that usually circulates between the GTP-bound active state and GDP-bound inactive state, which is partly promoted by the GTP hydrolysis-stimulating GTPase activation protein (GAP). However, when the RAS protein is mutated, impaired GAP stimulation promotes the formation of a persistently GTP-bound RAS. **(D)** An overview of the general biochemical destruction of hydrolysis and guanine exchange after mutation of codon 12 or 61.

RAS mutations may represent the early onset of tumorigenesis and are essential for tumor maintenance, which has been validated by considerable evidence (15–17). The RAS mutation rates in various cancer types are shown in **Supplemental Figure 1**. Different single-base missense mutations result in different amino acid substitutions of the RAS oncogene (**Figure 1A**). KRAS, HRAS, and NRAS are the three most commonly mutated RAS isoforms with varying mutation rates in different cancers (18). KRAS mutations, more than 80% of which are G12 mutations, are frequently found in pancreatic ductal adenocarcinoma (> 90%), colorectal adenocarcinoma (> 40%), and lung adenocarcinoma (approximately 30%). NRAS mutations, which occur less frequently than KRAS mutations, mainly occur at codon 61 and are found in nearly 30% of cutaneous skin melanomas. HRAS mutations occurring at codon 12 or 61 are only found in a small subset of bladder urothelial carcinoma, head and neck squamous cell carcinoma, and thyroid carcinoma (19–21). The top ten predominant substitutions and frequencies with which they occur in the three RAS isoforms according to tissue type in common cancers are shown in **Figure 1B**. For pancreatic ductal adenocarcinoma and colorectal adenocarcinoma, the predominant amino acid substitution is G12D in KRAS. For lung adenocarcinoma, the predominant amino acid substitution is G12C in KRAS. However, for melanoma, the predominant substitution is Q61R in NRAS.

As the most frequently mutated isoform of the RAS family, KRAS has two splice variants, KRAS4A and KRAS4B, which differ in their fourth exon and encode two different proteins that differ only in their C-terminal membrane-targeting regions (22, 23). KRAS4B is the main mutant isoform in human cancer, whereas KRAS4A is commonly expressed in various cancer cell lines and colorectal cancer (24, 25). Certain mutations in the amino acid sequence of KRAS often result in distinct transformation properties and biological behaviors (26). For instance, KRAS-G12V mutations are associated with worse outcomes than KRAS-G12D mutations in patients with lung cancer. Over the last 30 years, the correlation between biological behavior and specific RAS mutations has remained unclear (27–29). KRAS mutations are significantly associated with poor outcomes in patients with lung cancer (30, 31). However, a recent study suggested that for stages I-III, there was no statistical difference in overall survival (OS) between the mutant- and wild-type-carrying patients with NSCLC (32).

## 3 Domains and regions of KRAS

The RAS protein, cycling between inactive and active GDP-bound conformations, comprises three major domains: G-domain, C-terminal, and C-terminal CAAX motifs (33, 34). The G-domain is a highly conserved domain that includes switches I and II, which are responsible for the GDP-GTP exchange (33). The C-terminal region containing the CAAX motif varies considerably among different members of the RAS family. However, this motif is essential for post-translational modification (35). RAS is activated by guanine nucleotide exchange factors (GEFs) and transduces signals to downstream pathways.

KRAS encodes a membrane-bound GTPase that is inactive when bound to GDP and active when bound to GTP. The transition of KRAS to its active state is facilitated by GEFs such as SOS1. Once activated by extracellular stimuli, the active form of KRAS acts as a cellular switch, triggering downstream signaling pathways involved in fundamental cellular processes. Mutations in RAS block the binding of GTP to RAS and cause aberrant activation of downstream pathways (**Figure 1C**). RAS mutations may affect the intrinsic GTPase and GDP–GTP exchange rates (**Figure 1D**) (36). Mutations in KRAS at codons 12, 13, and 61 inhibit the ability of GTPase activation protein (GAP) to stimulate GTP hydrolysis. However, KRAS-G13D displays heightened intrinsic exchange activity compared to the wild-type RAS protein (37, 38). Despite the reduced p120 GAP-mediated hydrolysis rate, KRAS-G12C mutant exhibits almost wild-type intrinsic GTPase activity and has been used to develop covalent inhibitors (39).

## 4 KRAS inhibitors for patients with cancer

Despite over three decades of intensive efforts, no effective regimen to inhibit RAS-driven oncogenesis has been developed because of its inaccessible binding surface and picomolar affinity for GTP/GDP (7, 40). The high affinity of the RAS for cytoplasmic GTP renders competitive inhibition difficult to achieve. The absence of a drug-binding groove on the smooth surface of the RAS poses a challenge for targeted inhibitors. Multiple upstream and downstream regulators of RAS pathways contribute to drug resistance mechanisms and bypass signals, further limiting the effectiveness of combination strategies (41).

**Figure 2:**
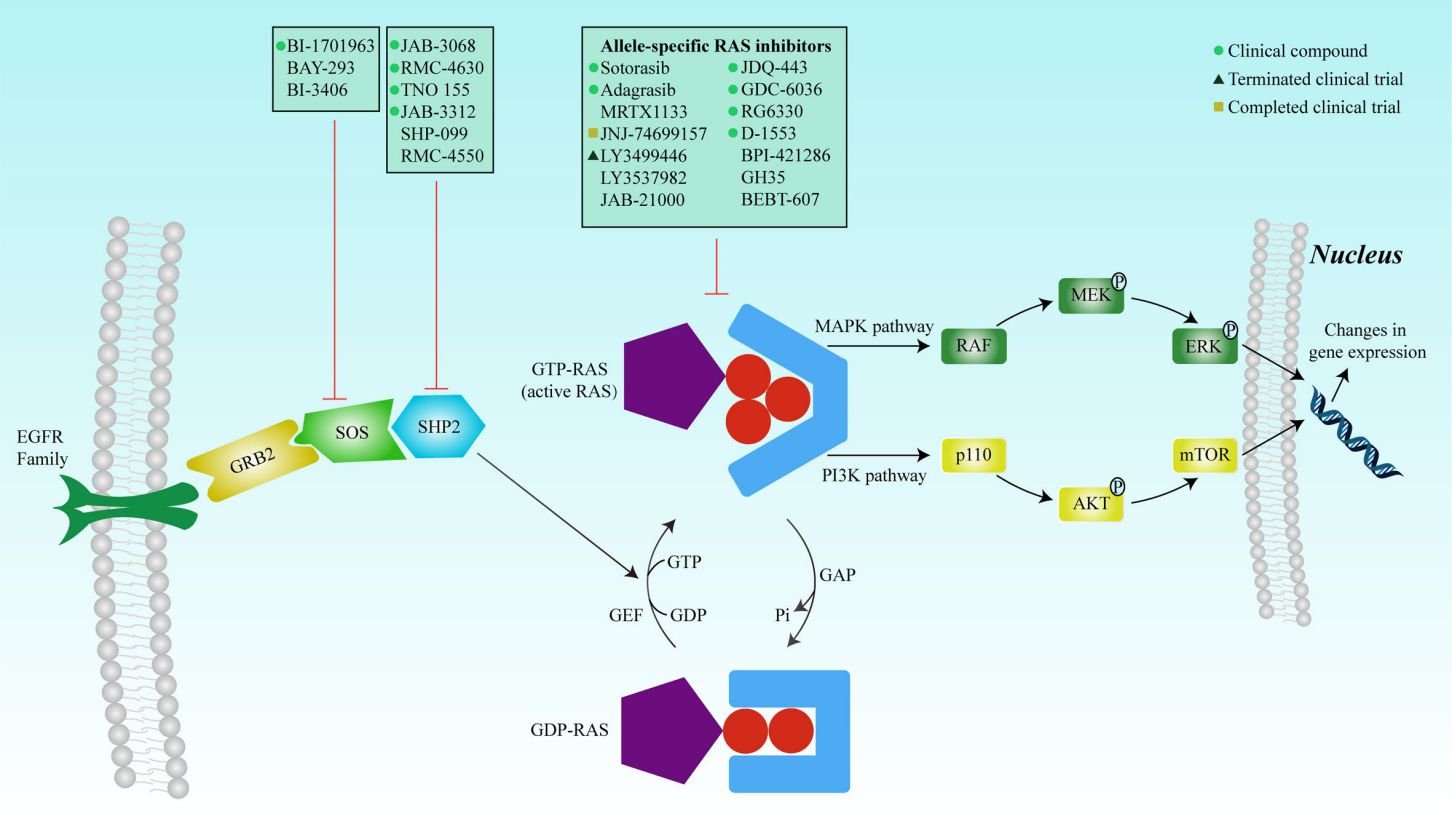
RAS mutation activates the protein, and the complex formed with GTP binds to the Ras-binding domain of the effector protein (RAF, PI3K, and RALGDS) to activate the MAPK and PI3K signaling pathways, respectively. The signals are transduced into the nucleus to regulate gene expression, thereby affecting cell proliferation and survival. Inhibition of SOS or SHP2 reduces the exchange rate between GDP and GTP and reduces the GTP-bound RAS population. Mutated RAS proteins accumulate in the GTP-bound state. Many inhibitors have been developed to directly inhibit RAS, including covalent allele-specific inhibitors that bind to KRAS-G12C.

These complexities underscore the challenges in targeting RAS mutations. In 2013, with the identification of a new covalent pocket of the KRAS-G12C mutation located beneath the effector-binding switch-II region, Shokat et al. reported a novel strategy for overcoming these difficulties in a mutant-specific targeting manner (42). A series of small-molecule agents could irreversibly bind to the KRAS-G12C mutation and disrupt switch-I and switch-II to bind the mutation in the GDP-bound state, thereby blocking the association with Raf and other downstream tyrosine kinases (**Figure 2**).

### 4.1 Sotorasib (AMG510)

Sotorasib (AMG510) is an oral small-molecule inhibitor that specifically and irreversibly inhibits the KRAS-G12C mutation (43). A preclinical study showed that sotorasib potently impaired the viability of two KRAS-G12C mutant cell lines NCI-H358 and MIA PaCa-2. Xenograft models have shown that AMG-510 can induce the regression of KRAS-G12C mutant tumors (9). The CodeBreak 100 phase I/II clinical trial evaluated the efficacy, safety, tolerability, and pharmacokinetics of sotorasib in patients with KRAS-G12C-mutant solid tumors (10). Of the 129 patients who participated in the phase I cohort study, 73 (56.6%) experienced mainly low-grade adverse events related to treatment (10). No treatment-related death or dose-limiting toxic effects were observed. The objective response rates (ORR) were 32.2% and 7.1% in NSCLC and colorectal cancer, respectively, indicating a promising anti-tumor activity for sotorasib in NSCLC.

The phase II cohort-based study revealed that out of 126 individuals diagnosed with advanced KRAS-G12C-mutant NSCLC, sotorasib treatment resulted in confirmed ORR and disease control rates (DCR) of 37.1% and 80.6%, respectively. The median response time during treatment was 10 months (44). A phase III clinical trial designed to compare the efficacy of sotorasib when administered alone versus docetaxel administration in previously treated patients with KRAS-G12C-mutant NSCLC is ongoing (NCT04303780). The progression-free survival (PFS) times for sotorasib-treated cohort were significantly higher than those of docetaxel-treated cohort (p=0.0017); a more favorable safety profile was also observed (45). Currently, sotorasib (AMG510) is approved by the FDA as a second-line treatment for patients with KRAS-G12C-mutant NSCLC who have received at least one systemic therapy (13, 14).

### 4.2 Adagrasib (MRTX849)

Adagrasib (MRTX849) is a KRAS-G12C inhibitor (46). According to preclinical studies, adagrasib effectively and consistently blocks KRAS-dependent signaling pathways with long-lasting effects, resulting in substantial tumor regression in 17 out of 26 (65%) KRAS-G12C-positive cell line- and patient-derived xenograft models (47). In a phase I/II clinical study, the KRYSTAL-1 trial evaluated the safety, tolerability, and clinical activity of adagrasib in patients with advanced solid tumors and a KRAS-G12C mutation (NCT03785249). Preliminary results showed that adagrasib monotherapy exhibited promising clinical activity and an acceptable safety profile in pretreated patients with advanced solid tumors (48). Particularly, for patients with NSCLC, among 51 patients evaluated for its clinical activity, ORR was 45% (23/51) and DCR was 96% (49/51). According to the present data from Mirati Therapeutics, patients with NSCLC with active brain metastases experienced a 63% reduction in the size of the primary lesion, and some lesions even disappeared after several cycles of adagrasib monotherapy. Among 18 patients with colorectal cancer, the ORR and DCR were 17% (3/18) and 94% (17/18), respectively. Interestingly, some coexisting mutations, including those in TP53, STK11, and KEAP1, may influence the efficacy of this anti-tumor agent. Preliminary results of KRYSTAL-1 showed that patients with advanced NSCLC and co-mutations of KRAS-G12C and STK11 had an ORR of 64% (9/14) across the pooled cohorts of phase I/Ib and II studies. A phase III study evaluating the efficacy of adagrasib versus docetaxel in previously treated patients with metastatic NSCLC and KRAS-G12C mutation is ongoing (NCT04685135).

### 4.3 Other KRAS inhibitors

Another KRAS inhibitor MRTX1133 selectively and reversibly inhibits KRAS-G12D and is currently being investigated in investigational new drug (IND)-enabling studies. Preclinical models have demonstrated the selective inhibition of cell viability in KRAS-G12D mutant tumor cells with a long predicted half-life (~50 h) (48). JNJ-74699157 (ARS-3248), a new-generation KRAS-G12C inhibitor, was developed based on ARS-1620 (11). A phase I clinical trial to determine the preliminary anti-tumor activity and safety in patients with advanced solid tumors and KRAS-G12C mutation showed that no significant clinical benefit was observed, with the best response to stable disease in four patients (40%). Moreover, an unfavorable safety profile prevented further enrollment and clinical development (49). The ARS-853 is a version of the ARS-1620 (50). Although they both inhibit cell growth and downstream signaling of the MAPK pathway in KRAS-G12C mutant tumor cell lines, ARS-853 is not suitable for use in animal models because of its lack of chemical and metabolic stability (11). JDQ-443 is another KRAS-G12C inhibitor currently in phase Ib/II clinical trial that evaluates the safety and tolerability of monotherapy in combination with other treatment drugs (spartalizumab and TNO155) in patients with advanced solid tumors and KRAS-G12C mutation (NCT04699188). Other KRAS-G12C inhibitors, GDC-6036 (NCT04449874), RG6330, and D-1553 (NCT04585035), are under phase I/II clinical trials, and their results have not been published. BPI-421286, GH35, BEBT-607, and JAB-21000, are all the KRAS inhibitors used in IND-enabling studies (51). The clinical developments of single-agent RAS inhibitors are summarized in **Table 1**.

**Table 1:** RAS inhibitor single agents and combination therapy in clinical development.

Drugs	Targets	ClinicalTrials.gov Number	Disease	Study Phase	Status	Interventions
Adagrasib (MRTX849)	KRAS-G12C	NCT03785249	Advanced solid tumors	I/II	Recruiting	Adagrasib
		NCT04685135	Advanced NSCLC	III	Recruiting	Adagrasib vs Docetaxel
MRTX1133	KRAS-G12D	/	/	/	Preclinical study	
JNJ-74699157 (ARS-3248)	KRAS-G12C	NCT04006301	Advanced solid tumors	I	Completed	JNJ-74699157
LY3499446	KRAS-G12C	NCT04165031	Advanced solid tumors	I/II	Terminated	LY3499446
LY3537982	KRAS-G12C	/	/	/	Preclinical study	
JDQ-443	KRAS-G12C	NCT04699188	Advanced solid tumors	Ib/II	Recruiting	JDQ443
GDC-6036	KRAS-G12C	NCT04449874	Advanced solid tumors	I	Recruiting	GDC-6036
RG6330	KRAS-G12C	/	/	I	Recruiting	RG6330
D-1553	KRAS-G12C	NCT04585035	Advanced solid tumors	I/II	Recruiting	D-1553
BPI-421286	KRAS-G12C	/	Advanced solid tumors	/	IND study	
GH35	KRAS-G12C	/	Advanced solid tumors	/	IND study	
BEBT-607	KRAS-G12C	/	NSCLC and colorectal cancer	/	IND study	
JAB-21000	KRAS-G12C	/	Advanced solid tumors	/	IND study	
Combination therapy of RAS inhibitor						
Sotorasib (AMG510)	KRAS-G12C	NCT03600883	Advanced solid tumors	I/II	Active	Sotorasib+PD-1/PD-L1 inhibitor
Adagrasib (MRTX849)	KRAS-G12C	NCT04613596	Advanced NSCLC	II	Recruiting	Adagrasib+ Pembrolizumab
		NCT03785249	Advanced solid tumors	I/II	Recruiting	Adagrasib+ Pembrolizumab/Afatinib (advanced NSCLC) Adagrasib+Cetuximab (Colorectal cancer)
		NCT04330664	Advanced solid tumors	I/II	Active	Adagrasib+TNO155
		NCT04793958	Colorectal cancer	IIII	Recruiting	Adagrasib+Cetuximab
LY3499446	KRAS-G12C	NCT04165031	Advanced solid tumors	I/II	Terminated	LY349944+ Abemaciclib/ Cetuximab/Erlotinib/Docetaxel
JDQ443	KRAS-G12C	NCT04699188	Advanced solid tumors	I/II	Recruiting	JDQ443+TNO155/ Spartalizumab/TNO155+ Spartalizumab
GDC-6036	KRAS-G12C	NCT04449874	Advanced solid tumors	I/II	Recruiting	GDC-6036+Atezolizumab (NSCLC) GDC-6036+Cetuximab (Colorectal cancer) GDC-6036+Bevacizumab (Advanced solid tumors) GDC-6036+Erlotinib (NSCLC)
D-1553	KRAS-G12C	NCT04585035	Advanced solid tumors	I/II	Recruiting	D-1553+other

With the development of new small-molecule inhibitors, previously undruggable mutant KRAS could be targeted. However, the complexity of the RAS pathway makes the treatment of RAS-mutant tumors challenging. The heterogeneity of the response to the same KRAS inhibitor among different tumor types forces researchers to consider the difference in the same mutation isoform in downstream signaling pathways and the feedback effects of the various tumors (52), as not only are cells intrinsic factors but the tumor microenvironment, particularly inflammation, also has the potential to modify susceptibility to oncogenic RAS mutations. It has been observed that certain cells can have an anti-neoplastic response against oncogenic RAS due to the activation of tumor suppressor pathways, while others cannot. The role of cell lineage in this response is of significant importance (53). Moreover, one possible explanation for the heterogeneity is the existence of different signaling dependencies in different tumor types. While some tumors heavily rely on KRAS signaling for growth and survival, others may have acquired alternative signaling pathways to compensate for KRAS inhibition. These alternative pathways can bypass the need for KRAS signaling, rendering the KRAS inhibitor less effective. Moreover, the co-occurring genetic alterations in different tumor types can contribute to the heterogeneity of response (54).

In addition, most KRAS inhibitors have been developed to target the KRAS-G12C mutation, which constitutes only a portion of the KRAS mutations and is commonly found in lung cancer (55). Therefore, new approaches are warranted to effectively treat other KRAS mutations such as KRAS-G12D and KRAS-G12V.

## 5 Evidence for pan-RAS inhibitors in RAS-mutant cancers

Although covalent inhibitors that directly target specific KRAS mutations exhibit promising efficacy, inhibiting other RAS mutations is challenging. New inhibitors have been developed, regardless of the type of RAS mutation or protein. A multivalent small molecular inhibitor compound 3144 was designed to interact with adjacent sites on the KRAS surface and disrupt interactions between RAS proteins and their effectors (56). Preclinical models showed that compound 3144 was capable of binding to HRAS, KRAS, and NRAS and inhibited RAS signaling. Xenograft models also indicated that 3144 could prevent the growth of RAS-mutant mouse cancer xenografts derived from tumor cell lines and patients. Satchell et al. developed a pan-RAS biologic inhibitor by fusing the RAS-RAP1-specific endopeptidase to the diphtheria toxin, which could irreversibly cleave and inactivate intracellular RAS at picomolar concentrations and terminate downstream signaling and induce tumor shrinkage in mouse xenograft models driven by either wild-type or mutant RAS (57). Furthermore, a compound named cmp4 selectively binds to the Switch II pocket of both HRAS and KRAS proteins with different mutations. By interfering with the binding of RAS to GEFs and Raf effectors, cmp4 effectively reduced the intrinsic and GEF-mediated nucleotide dissociation and exchange processes of the Ras protein, ultimately leading to the inhibition of the mitogen-activated protein kinase signaling pathway and a decrease in cell viability. According to a mathematical model of the RAS activation cycle, cmp4 when combined with cetuximab reduces the proliferation of cetuximab-resistant cancer cell lines. However, the affinity of cmp4 for RAS is unsatisfactory, and this limits its application as an ideal clinical drug (58).

Unfortunately, all these compounds that could function as pan-RAS inhibitors have only been tested in preclinical studies. Given the essential role of RAS in normal cell signaling, it is unclear whether pan-RAS inhibitors are tolerated. Previous studies have revealed that homozygous deletion of KRAS is embryonically lethal in mice (59–61). Therefore, the toxicity of pan-RAS inhibitors should be investigated in future studies. In addition, acquired resistance to RAS inhibitors often prevents further clinical benefits. Awad et al. compared the genomic and histological landscapes of pretreatment samples and those obtained after the development of resistance. Acquired KRAS alterations included G12D/R/V/W, Q61H, R68S, and high-level amplification of the KRAS-G12C allele. Bypass mechanisms involve MET amplification, mutations in NRAS and BRAF, and the oncogenic fusion of ALK and RET. Loss-of-function mutations in NF1 and PTEN have been previously reported. Consequently, new therapeutic strategies are necessary to overcome and delay drug resistance in patients with cancer (62).

## 6 KRAS mutations and immune landscape

Specifically, mutant KRAS not only alters the behavior of cancer cells but also affects various cells in the tumor microenvironment (TME). KRAS activation increases the production of the neutrophil chemokines CXCL1, CXCL2, and CXCL5 (63). The upregulation of intercellular adhesion molecule 1 (ICAM1) by KRAS promotes the recruitment of pro-inflammatory M1 macrophages (in contrast, co-activation of KRAS and MYC increases the recruitment of anti-inflammatory M2 macrophages by releasing CCL9 and IL-23). KRAS-mediated secretion of TGFβ and IL-10 leads to the differentiation of immunosuppressive regulatory T cells (Tregs). It also enhances tumor-infiltrating myeloid-derived suppressor cells (MDSCs) through GM-CSF-dependent and IRF2/CXCL3-dependent mechanisms (64).

Moreover, different co-mutation statuses of KRAS can affect the TME and response to immune checkpoint inhibitors (ICIs). For example, tumors with KRAS/STK11 co-mutations often exhibit deficiencies in CD8+ T lymphocytes and a high abundance of T-regulatory cells in the microenvironment. In contrast, tumors with KRAS/p53 co-mutations tend to have an inflamed TME characterized by a higher number of CD8+ T lymphocytes. This can be attributed to p53 mutations, which tend to increase somatic tumor mutations and potentially lead to the development of tumor neoantigens (65).

A detailed understanding of these pleiotropic effects will facilitate the rational design of curative combination therapies. Leidner et al. reported a patient with metastatic pancreatic cancer who received a single infusion of genetically engineered autologous T-cells targeting mutant KRAS-G12D. This led to a 72% partial response at 6 months according to the currently ongoing Response Evaluation Criteria in Solid Tumors version 1.1. Engineered T cells constitute over 2% of the circulating T cells (66). The occurrence of distinct co-mutations affects the clinical efficacy of immunotherapies. In another study involving 536 patients with KRAS-mutant lung adenocarcinoma, both STK11 and KEAP1 mutations in the presence of a KRAS mutation were associated with poor response rates to anti-PD-L1 inhibitors. Median PFS and OS were significantly shorter for KRAS-mutant/STK11-mutant NSCLC (2.0 and 6.2 months, respectively) than that for KRAS-mutant/STK11-wildtype (4.8 and 17.3 months, respectively; HR 2.04, 95% CI 1.66–2.51, p < 0.0001) varieties. Similarly, patients with KRAS-mutant/KEAP1-mutant NSCLC had lower PFS and OS (1.8 and 4.8 months, respectively) than those with KRAS-mutant/KEAP1-wildtype variety (4.6 and 18.4 months, respectively; HR 2.05, 95% CI 1.63–2.59, p < 0.0001) (67).

### 6.1 Immunotherapy in KRAS-mutant cancers

Immunotherapy has revolutionized the landscape of cancer therapy, especially ICIs, which have been aggressively tested in almost all cancer types. The discovery of immune checkpoints, including cytotoxic T lymphocyte protein 4 (CTLA4), PD-1, and PD-L1, was a breakthrough in cancer immunotherapy. Data obtained from human cancer studies and transgenic mouse models suggest that immune responses aimed at safeguarding the host can be overcome in RAS-driven cancers (47). A KRAS-G12D-induced mouse model also demonstrated that the initial immune response was inhibited, eventually leading to immune evasion. Therefore, resuscitation of the depressed immune surveillance system may be an efficient approach for the treatment of RAS-mutant cancers.

A good immunotherapy response is predicted by a high mutational burden, elevated PD-L1 expression, and an increased prevalence of tumor-infiltrating lymphocytes (TILs). KRAS-mutant NSCLC cells display a high mutational burden and are densely infiltrated by T-cells. In addition, a meta-analysis of 26 studies (n=7,541 patients) indicated that tumors with KRAS mutations had higher levels of PD-L1 than tumors without KRAS mutations; odds ratio (OR) =1.45, 95% CI, 1.18-1.80, P= 0.001) (68). Further, KRAS mutations can induce the upregulation of PD-L1. According to Coelho et al., PD-L1 expression in tumor cells can be influenced by activating the oncogenic RAS pathway, which is accomplished through post-transcriptional regulation of PD-L1 mRNA (69).

Thus, immunotherapy for KRAS-mutant lung cancer may show potential. A subgroup analysis of CheckMate-057 exhibited prolonged outcomes with ICIs than with docetaxel in patients with KRAS-mutant NSCLC (mean OS, 12.2 vs 9.4 months; P=0.002) (70). The exploratory analysis of KEYNOTE-042 revealed pembrolizumab monotherapy as the first-line therapy, which exhibited more pronounced benefits over chemotherapy in patients with KRAS mutations (mean OS, 28 vs 11 months; hazard ratio, 0.42; 95% CI, 0.22-0.81) than those with KRAS wild type (mean OS, 15 vs 12 months; hazard ratio, 0.86; 95% CI, 0.63-1.18). Recently, a retrospective study evaluated the correlation of KRAS status with outcomes following immunotherapy in patients with PD-L1≥50%. Among patients treated using ICI monotherapy, the KRAS variant was related to a superior survival than did KRAS wild-type (mean OS, 21.1 vs 13.6 months; P =0.03). The CCTG PA.7 study compared gemcitabine and nab-paclitaxel, with and without durvalumab and tremelimumab, in metastatic pancreatic ductal adenocarcinoma. Combination immunotherapy did not improve survival among the unselected patient population but improved survival for patients with wild-type KRAS tumors (NCT02879318) (71).

Many patients with KRAS-mutant NSCLC receive ICIs as first-line treatment because of their limited approval for second-line use. Combining KRAS inhibitors with ICIs is logical given the diverse mechanisms of mutant KRAS during immune response. Mouse models treated with sotorasib and ICIs showed pro-inflammatory changes in the TME and synergistic tumor cell killing. Adagrasib also induces a pro-inflammatory state and enhances immune cell infiltration. Combination therapy resulted in lasting anti-tumor and memory immune cell responses in mice. Future studies should explore combination therapies, predictive biomarkers, and mechanisms of resistance in KRAS-mutant cancers (9).

## 7 Combination therapy of RAS inhibitors for RAS-mutant cancers

In preclinical models, combination treatment with AMG510 caused regression of KRAS-G12C-mutant tumors and improved the anti-tumor efficacy of targeted agents and chemotherapy (9). When combined with immunotherapy, AMG510 induces complete and durable tumor regression. The improved efficacy of the combination therapy may be attributed to increased immune cell infiltration and activation. In preclinical models, the AMG510 monotherapy and combination therapy groups demonstrated a notable increase in CD8+ T cell infiltration, which was not observed in the anti-PD-1 monotherapy group. Additionally, AMG510 treatment increased the infiltration of macrophages and CD103+ cross-presenting dendritic cells, which play vital roles in T-cell priming, activation, and recruitment. Furthermore, the combination of AMG510 and anti-PD-1 therapy promoted the establishment of a memory T cell response and enhanced antigen recognition. Phase I/II clinical trials evaluating the efficacy and safety of sotorasib in combination with PD-1/PD-L1 inhibitors in patients with advanced solid tumors and KRAS-G12C mutations are ongoing (CodeBreaK 100/101).

Preclinical models have also demonstrated that human epidermal growth factor receptor (EGFR) family inhibitors, SHP2 inhibitors, mammalian target of rapamycin (mTOR) inhibitors, and inhibition of CDK4/6 could enhance the anti-tumor activity of MRTX849 and inhibit KRAS-dependent signaling pathways (46). Clinical trials were conducted to evaluate the efficacy and safety of combination therapy of adagrasib with pembrolizumab (a PD-1 inhibitor) or afatinib (an HER family inhibitor) in patients with NSCLC, with cetuximab in patients with colorectal cancer, and with TNO-155 in patients with advanced solid tumors. Preliminary results showed that more than 50 patients were treated with adagrasib in combination with either pembrolizumab (a PD-1 inhibitor) for NSCLC, cetuximab (an anti-EGFR antibody) for colorectal cancer, or TNO-155 (an SHP-2 inhibitor) for NSCLC or colorectal cancer. All the combination therapies were well tolerated by patients (48). A phase I-II clinical trial evaluated the efficacy and safety of adagrasib monotherapy or in combination with cetuximab in heavily pretreated patients with metastatic colorectal cancer and mutant KRAS-G12C. The results revealed that 19% of the 43 evaluated patients in the monotherapy group responded, with a median response duration of 4.3 months and a median PFS of 5.6 months. However, the combination therapy group had a higher response rate (46%), with a median response duration of 7.6 months and a median PFS of 6.9 months (72). A phase II clinical trial evaluated the efficacy of adagrasib in patients with KRAS-G12C-mutant NSCLC previously treated with platinum-based chemotherapy and anti-PD-1 or PD-L1 therapy. The results showed that 48 of the 112 enrolled patients had a confirmed objective response, with a median response duration of 8.5 months and a median PFS of 6.5 months. The median OS was 12.6 months (73).

A phase Ib/II clinical trial to characterize the safety and tolerability of JDQ443 in combination with TNO155, spartalizumab (a PD-1 inhibitor), or TNO155 and spartalizumab in patients with advanced solid tumors and KRAS-G12C mutations is ongoing (NCT04699188) (74). Another phase I trial to assess the safety and preliminary activity of GDC-6036 in combination with atezolizumab (a PD-L1 inhibitor) or erlotinib in patients with NSCLC, cetuximab in patients with colorectal cancer, or bevacizumab in patients with advanced solid tumors is underway (NCT04449874). D-1553 is also the regimen used in clinical trials to assess the anti-tumor activity of combination therapy of RAS inhibitors with other treatments (NCT04585035). However, the results of these studies have not been reported. The combination therapies for RAS inhibitors used in clinical development are shown in **Table 1**.

## 8 Inhibitors of KRAS and associated molecular pathways

### 8.1 Upstream RAS pathways and KRAS inhibitors

Normal RAS upstream signaling requires activation by GEFs, membrane localization, effector binding, and nucleotide exchange and processing (75). Therefore, the disruption of any of these steps could indirectly inhibit RAS activation. Son of Sevenless (SOS) is a GEF that activates important cell signaling pathways and acts as a pacemaker for the RAS (76). Elimination of SOS1 specifically induces a decrease in the survival rate of tumor cells carrying a KRAS mutation, while exhibiting no significant impact on those with wild-type KRAS (77). BAY293, BI-3406, and BI-1701963 are SOS1 inhibitors developed to inhibit the protein-protein interactions of KRAS-SOS1 (78–80). However, preclinical studies have shown that BAY 293 only demonstrates modest antiproliferative effects, and no significant difference between KRAS mutation and wild-type was observed (78). BI-3406 exhibited more encouraging anti-tumor activity. It not only selectively inhibited the proliferation of KRAS-mutant cancer cells but also blocked the negative feedback reactivated by SOS1 (79). BI-1701963, an improved version of BI-3406, is currently in three phase I trials to determine the safety, tolerability, and pharmacokinetic parameters of BI-1701963 monotherapy or in combination with trametinib, BI-3011441 (a MEK inhibitor), or irinotecan in patients with KRAS-mutated cancers (NCT04111458, NCT04835714, and NCT04627142).

As a non-receptor protein tyrosine phosphatase, SHP2 is encoded by PTPN11, plays an important role in signal transduction downstream of various growth factors, and increases RAS nucleotide exchange by binding to GRB2 and SOS1 (81). The complete activation of the RAS-MAPK pathway requires SHP2; thus, the essential role of SHP2 in oncogenic signaling is established. The inhibition or deletion of SHP2 delays tumor progression in established tumors. SHP-099 and RMC-4550 are both potent and selective SHP2 allosteric inhibitors (82, 83). Both reduced cell proliferation, but the sensitivities differed among different KRAS-mutated cancer cells. Another study revealed that IACS-13909, a potent and specific allosteric inhibitor of SHP2, effectively inhibited tumor cell proliferation *in vitro* and caused regression of tumors *in vivo* in NSCLC models that exhibited resistance to osimertinib due to EGFR mutations (84). However, the anti-tumor activity of IACS-13909 against KRAS-mutant cancer cells has not yet been established.

Although SHP2 inhibitors offer a potential therapeutic solution for receptor tyrosine kinase-driven cancers, they may not adequately suppress tumor growth in KRAS-mutated cells when administered alone (83). In KRAS-mutant tumors, resistance to MEK inhibition is common owing to the activation of the receptor tyrosine kinase signaling pathway. However, combination treatment with MEK and SHP2 inhibitors resulted in the continued regression of tumor growth in xenograft models of pancreatic cancer and NSCLC derived from patients, indicating the clinical efficacy of dual SHP2/MEK inhibition for KRAS-mutant cancers (85).

RMC-4630 (SAR442720) is an SHP2 inhibitor under phase I/II trial that evaluates the safety, MTD, and RP2D of RMC-4630 in combination with cobimetinib in patients with relapsed/refractory solid tumors and combination with osimertinib in patients with EGFR-mutant locally advanced or metastatic NSCLC (NCT04000529). Another two phase I trial evaluating the safety of RMC-4630 monotherapy (NCT03634982) and in combination with pembrolizumab (NCT04418661) in advanced solid tumor patients presented in the AACR ANNUAL MEETING 2020 showed that the combination of RMC-4630 with cobimetinib has acceptable tolerability, and tumor reduction was observed in three of eight patients with KRAS-mutant colorectal cancer, including one unconfirmed PR at the data cut-off (86). TNO155 (NCT03114319, NCT04000529, NCT04330664, and NCT04699188), JAB-3068 (NCT04721223, NCT03518554, and NCT03565003), and JAB-3312 (NCT04121286 and NCT04045496) are all SHP2 inhibitors currently in clinical trials. However, the results of these studies have not yet been published.

In addition, complete RAS activation requires a post-translational process to associate with the membrane, protein oligomerization or dimerization, and effector binding. RAS can also self-associate to enhance scaffolding and signaling activities via dimerization. Disruption of any of these steps appears to effectively block RAS signaling. However, there remains a challenge that needs to be overcome. Enzymes involved in the post-translational process also process other membrane-associated proteins that can cause intolerable toxicity. Owing to the challenges in reconstituting RAS dimers and oligomers *in vitro*, the study of the molecular intricacies of RAS-RAS interactions has been limited to a combination of computational modeling and experimental validation of protein interactions.

### 8.2 Downstream effectors of RAS pathways and KRAS inhibitors

Once activated, RAS interacts with a diverse array of downstream effectors, each of which plays a unique role in signal transduction. Some key effector pathways include the RAF-MEK-ERK, PI3K-AKT-mTOR, and RalGDS pathways. The RAF-MEK-ERK pathway is one of the most well-studied RAS effector pathways. It involves the activation of RAF kinases (such as ARAF, BRAF, and CRAF), which phosphorylate and activate MEK1/2. MEK1/2 then phosphorylates and activates ERK1/2, leading to the regulation of gene expression and cellular processes, such as proliferation, differentiation, and survival. The PI3K-AKT-mTOR pathway is an important RAS effector pathway. RAS activates phosphoinositide 3-kinase (PI3K), leading to the production of phosphatidylinositol-3,4,5-trisphosphate (PIP3). PIP3 recruits and activates protein kinase B (AKT), which regulates multiple downstream effectors involved in cell growth, metabolism, and survival. AKT also regulates the mammalian target of the rapamycin (mTOR) pathway, thereby influencing protein synthesis and cell proliferation. The RalGDS pathway involves the activation of the Ral guanine nucleotide dissociation stimulator (RalGDS) by RAS. RalGDS activates Ral GTPases that participate in diverse cellular processes, including cytoskeletal organization, membrane trafficking, and cell transformation. These downstream effectors represent only a fraction of the intricate network of signaling pathways regulated by RAS. The complexity and diversity of RAS signaling indicate its fundamental importance in cellular physiology and its role in various diseases, particularly cancer (87, 88).

Downstream effectors of the RAS pathway, particularly those in the RAF-MEK-ERK and PI3K-AKT-mTOR signaling pathways, have become attractive targets for anti-RAS mutation treatment. Numerous inhibitors targeting different constituents of the RAF-MEK-ERK and PI3K-AKT-mTOR effector pathways have been developed and are currently undergoing clinical assessment; however, their effectiveness appears to be limited (89–91). The RAF pathway plays a significant role in the promotion of RAS-driven cancer growth. Studies conducted in mouse models have indicated that only the constituents of the RAF-MEK-ERK pathway can compensate for the loss of RAS function and revive the growth of RAS-deficient mouse embryonic fibroblasts. However, inhibition with a single-component RAF, MEK, or ERK could lead to negative feedback, which might explain poor efficacy (92). Although the PI3K pathway may have a minimal effect on promoting RAS-dependent cancer growth, it complements the RAF-MEK-ERK cascade. Therefore, resistance to RAF pathway inhibitors may be mediated via the PI3K pathway. Thus, a combination strategy with other inhibitors as mentioned previously or immunotherapy might be required to completely suppress the signaling pathway as an effective strategy for RAS-mutant cancer.

Although the clinical data of immunotherapy are limited in other solid tumors with RAS mutations, the efficacy of a combinational strategy of immunotherapy with RAS inhibitors or inhibitors of downstream effectors of the RAS pathway, particularly the MAPK pathway, is worth anticipating, and the possible reason has been discussed previously. Clinical trials are ongoing, as previously discussed. The adoptive cell approach and cancer vaccines, two other immunotherapeutic approaches to treat RAS-driven cancers, have shown certain efficacy, but further research is still needed (93, 94).

## 9 Discussion

KRAS mutations have long been considered attractive targets for cancer therapy. After decades of effort, KRAS mutations are no longer considered undruggable. KRAS-G12C allele-specific inhibitors exhibit promising efficacy in clinical trials and have the potential to alter the treatment status of RAS-mutant cancers. Sotorasib and adagrasib have shown promising results in inhibiting KRAS-G12C and controlling tumor growth. Disease control was observed in a significant percentage of patients, and tumor shrinkage was also noted. However, some patients developed resistance mechanisms, such as mutations activating RAS or the RAS pathway, which rendered the drugs less effective. Combining KRAS-G12C inhibitors with other targeted therapies, like cetuximab or SHP2 inhibitors, has shown enhanced activity in preclinical studies. Resistance mutations were more frequent in patients with lung or colorectal cancer treated with adagrasib. Multiple types of lesions were identified, including mutations preventing drug binding, non-G12C activation of RAS, KRAS amplification, and activation of other pathway components. The presence of multiple and diverse resistance mechanisms poses a challenge to the efficacy of RAS inhibitors. However, similar mechanisms have been observed in resistance to other targeted therapies, indicating the need for further investigation. Despite these challenges, KRAS-G12C inhibitors have demonstrated clinical benefit and are likely to be useful as second-line treatments for lung cancer. Continued research and development are expected to lead to improved drugs and combination therapies that can enhance tumor-cell death and prevent adaptive resistance. Additionally, a new G12C inhibitor that targets active RAS-GTP is being developed and has shown effectiveness against KRAS-G12C tumor cells with resistance to previous inhibitors.

Even though the inhibition of the RAS pathway, including the MAPK and PI3K pathways, showed poor efficacy after monotherapy, a combinational strategy could be useful to improve efficacy. Patients with KRAS-mutant NSCLC can benefit from immunotherapy, and clinical trials evaluating the efficacy of adoptive cell therapy and cancer vaccines are ongoing.

Agents inhibiting RAS post-translational modifications during development have also been researched. Posttranslational modifications of RAS proteins include palmitoylation and depalmitoylation. Palmitoylation attaches palmitic fatty acids to specific amino acid residues, thereby promoting membrane associations and functionality. Depalmitoylation removes these groups and redistributes RAS proteins to the active membrane sites. Inhibition of depalmitoylation has been proposed to hinder RAS membrane binding and functionality. Other modifications such as phosphorylation, nitrosylation, ubiquitination, and acetylation also regulate RAS localization and function. These modifications are potential targets for the development of anti-RAS drugs; however, their mechanisms of action and therapeutic relevance are still controversial. Further research is required to validate their feasibility and specificity for anticancer therapy (8).

Given the encouraging efficacy of KRAS-G12C allele-specific inhibitors, specific inhibitors may be the most promising therapeutic options. However, in addition to KRAS-G12C, other mutations, such as KRAS-G12D and KRAS-G12V, account for a large proportion of KRAS mutations. Therefore, the development of inhibitors targeting specific RAS mutations to provide personalized medicine may be a future direction. However, according to the presented results, the efficacy of sotorasib differs in NSCLC and colorectal cancer and drug resistance is inevitable (10, 52). In addition, combination therapies involving immunotherapy and other targeted therapies or chemotherapies may be worth exploring. The studies discussed in previous sections have shown promising outcomes when KRAS inhibitors were combined with ICIs or other targeted agents. Further investigations should focus on optimizing the treatment regimens, identifying predictive biomarkers, and understanding the mechanisms underlying the synergistic effects observed in preclinical models. Furthermore, understanding the TME and the role of immune cells in KRAS-mutant cancers is crucial. Exploring the factors influencing immune cell infiltration, activation, and recruitment can help in designing strategies to enhance anti-tumor immune responses. Investigating the mechanisms underlying immunotherapy resistance in KRAS-mutant cancers is an important area for future research. This knowledge can guide the development of novel therapeutic approaches to overcome drug resistance and improve patient outcomes. To address these unresolved issues, developing a comprehensive model that integrates the complex interactions between KRAS signaling, the immune system, and the tumor microenvironment would be valuable. Such a model could help explain the observed heterogeneity in treatment responses and potentially predict personalized treatment regimens and responses. This could also guide the design of clinical trials and treatment strategies. Therefore, exploring combination strategies for patients with distinct tumors is vital.

## Author contributions

XW drafted the manuscript. WS and CC helped in the literature search. DL provided administrative and financial support, manuscript revision, and final approval of the manuscript. All authors contributed to the article and approved the submitted version.
