# Supplementary figures and images for: Small molecular inhibitors for KRAS-mutant cancers
Source: Front Immunol. 2023 Aug 18;14:1223433. doi: 10.3389/fimmu.2023.1223433 (PMC10470052; doi:10.3389/fimmu.2023.1223433)

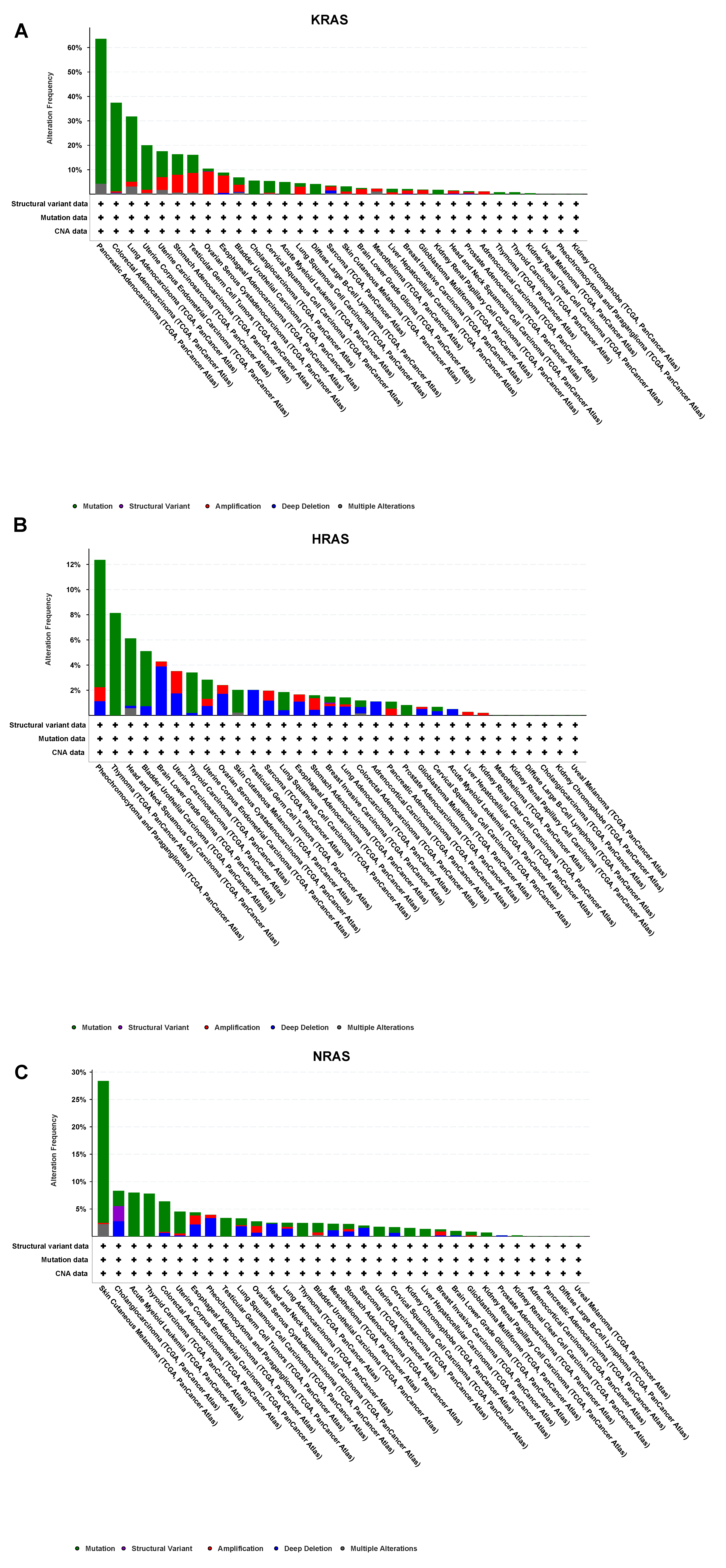

Supplement: Supplementary file 1 [file Image_1.tiff]
